# Hospicells promote upregulation of the ATP-binding cassette genes by insulin-like growth factor-I via the JAK2/STAT3 signaling pathway in an ovarian cancer cell line

**DOI:** 10.3892/ijo.2013.2017

**Published:** 2013-07-15

**Authors:** NADIA BENABBOU, PEZHMAN MIRSHAHI, MÉLODIE CADILLON, JEANNETTE SORIA, AMU THERWATH, MASSOUD MIRSHAHI

**Affiliations:** National Institute for Medical Research (INSERM), Cordeliers Research Center (UMRS 872), University of Pierre and Marie Curie and University of Paris Descartes, Paris, France

**Keywords:** insulin-like growth factor-I, JAK2-STAT3, MEK, PI3-kinase signaling pathway, ATP binding cassette, hospicells, chemoresistance, ovarian cancer

## Abstract

Interaction between tumor cells and their microenvironment has a crucial role in the development, progression and drug resistance of cancer. Our objective was to confirm the role of Hospicells, which are stromal cells from the cancer microenvironment, in drug resistance and tumor cell growth. We demonstrated that soluble factors secreted by Hospicells activate several genes and upregulate the JAK/STAT signaling pathway in ovarian cancer cell lines. Hospicells express all insulin-like growth factor (IGF) family as detected by gene array, RT-PCR, protein array and immunocytochemistry. While focusing attention on the microenvironment, we considered the role of IGF-I in proliferation and survival of ovarian cancer cells. Indeed, IGF-I is a major regulator of different stages of cancer development. We studied the effect of exogenously added IGF-I on the regulation of ATP-binding cassette (ABC) genes (MDR1, MRP1, MRP2, MRP3, MRP5 and BCRP) in the ovarian cancer cell line OVCAR3 and validated the results obtained using the IGF-IR antagonist picropodophyllin. IGF-I regulates the expression of ABC genes in OVCAR3 cells via the PI3-kinase, MEK and JAK2/STAT3 signaling pathways. The OVCAR3 cell line when co-cultured with Hospicells showed a marked degree of drug resistance. The drug resistance observed could be amplified with exogenous IGF-I. Addition of IGF-IR inhibitor, however, reduced the degree of resistance in these exposed cells. Cells that were treated with anticancer drugs and then exposed to IGF-I showed an increase in drug resistance and, thereby, an increase in cell survival. This observation indicates that drug resistance of OVCAR3 cells increases when there is synergy between OVCAR3 cells and Hospicells and it is amplified when IGF-I was exogenously added. In conclusion, inhibition of IGF-IR and targeting of the JAK2/STAT3 signaling pathway can be a target for ovarian cancer therapy.

## Introduction

The tumor microenvironment has been largely studied as a dynamic system orchestrated by several growth factor such as insulin like growth factor-I (IGF-I) (1–4). IGF-I functions by autocrine and/or paracrine effect, in physiological and pathological processes, but is also involved in development and progression of various cancers such as ovarian cancer (5). High levels of free IGF-I and IGF-I activity are associated with elevated risk of progression in ovarian cancer (6). In plasma, IGF-I binds mainly to the IGF binding protein, IGFBP-3 (7). Ovarian cancer is the sixth most frequent tumor in women and the first cause of death from the gynaecological cancers in the world (8). The efficiency of treatments is often hindered due to emergence of multi-drug resistance (MDR). The microenvironment surrounding the tumor plays a significant role in MDR development (9) and also in the progression and metastasis. Peritoneal homeostasis is influenced by growth factors and stromal cells. The peritoneal stromal cells present in ascitic fluids of ovarian cancer have been shown to stimulate cell growth (10,11). A distinctic population of stromal cells forming the tumor microenvironment of floating cell aggregates from ascitic fluid was identified and isolated for the first time in our laboratory and named ‘Hospicells’ (9,12–15). The Hospicells have been shown to confer drug resistance to cancer cells during chemotherapy by trogocytosis (cell-cell contact). At present we provide data demonstrating that IGF-I upregulates MDR1, MRP1, MRP2 and BCRP, ATP binding cassette gene family via PI3-kinase, MEK and JAK2/STAT3 pathway in OVCAR3 cells. Hospicells and ovarian cancer cells are mutually synergic in the presence of each other for the development of multi-drug resistance.

## Materials and methods

### Cell culture

#### Cell lines

The cell line of human ovarian adenocarcinoma (OVCAR3) was from the American Type Culture Collection (ATCC). OVCAR3-eGFP cells, genetically modified by lentiviral vector encoding eGFP (Genethon, Evry, France), were also used in this study. These two cell lines were maintained in culture using RPMI-1640 medium containing 10% fetal bovine serum (FBS), 1% L-glutamine and 50 U/ml antibiotics (penicillin and streptomycin) and incubated at 37°C in an atmosphere humidified with 5% CO_2_.

#### Hospicells isolated from ascites

The Hospicells were isolated from ascites of patients with ovarian cancer at stage III. These cells were obtained according to the protocol described by Rafii *et al*(9). These Hospicells were immortalized with T antigen of the SV-40 virus (P-BABE largeT SV40, Addgene, Cambridge, MA, USA) and were named M16 cells. The M16 cells were grown in RPMI-1640 medium at 37°C in 5% CO_2_.

#### Preparation of supernatant from Hospicells (M16 cells)

M16 cells (10^6^ cells/ml of culture medium) were incubated for 24 h at 37°C in RPMI without FBS. After incubation, the supernatant was collected and stored at −80°C. The supernatant was studied for its effect on resistance of OVCAR3 cells against chemotherapy drugs in the presence of IGF-I or in the presence of inhibitor of IGF-IR, called cyclolignan picropodophyllin (PPP).

#### Treatment of OVCAR3 cells with IGF-I and inhibitor of signaling pathways

The adherent OVCAR3 cells were incubated with 200 ng/ml of IGF-I (Promocell GmbH, Heidelberg, Germany) and the following inhibitors from Calbiochem (Paris, France): 1 μM of picropodophyllin (an inhibitor of IGF-IR); 5 μM of wortmannin (PI3 kinase inhibitor), 5 μM of rottlerin (PKC inhibitor); 5 μM of PD98059 (MEK inhibitor), 5 μM of Jak2 inhibitor and 5 μM of STAT3 inhibitor.

#### Reverse transcription and polymerase chain reaction (RT-PCR)

Total RNA was extracted with the Nucleospin RNA II kit (Macherey-Nagel EURL, Hoerdt, France). Reverse transcription was performed using M-MLV reverse transcriptase and oligo (dT) primers (Gibco-BRL, Paisley, UK). The polymerase chain reaction (PCR) was performed by Taq DNA polymerase (Gibco-BRL). Specific primers for: IGF-I (sense, 5′-AAA TCA GCA GTC TTC CAA C-3′; antisense, 5′-CTT CTG GGT CTT GGG CAT GT-3′); IGF-II (sense, 5′-AGT CGA TGC TGG CTT CTC A-3′; antisense, 5′-GTG GGC GGG GTCT TGG GTG GGT AG-3′); IGF-IR (sense, 5′-GAC ATC CGC AAC GAC TAT CAG-3′; antisense, 5′-GTA GTT ATT GGA CAC CGC ATC-3′); IGF-IIR (sense, 5′-TAC AAC TTC CGG TGG TAC ACC A-3′; antisense, 5′-CAT GGC ATA CCA GTT TCC TCC A-3′); MDR1 (sense, 5′-GTT ATA GGA AGT TTG AGT TT-3′; antisense, 5′-AAA AAC TAT CCC ATA ATA AC-3′); MRP (sense, 5′-AAT GCG CCA AGA CTA GGA AG -3′; antisense, 5′-ACG GGA GGA TGT TGA ACA AG-3′); MRP2 (sense, 5′-CTG GTT GAT GAA GGC TCT GA-3′; antisense, 5′-CTG CCA TAA TGT CCA GGT TC-3′); MRP3 (sense, 5′-GCA GGT GAC ATT TGC TCT GA-3′; antisense, 5′-CCC TCT GAG CAC TGG AAG TC-3′); MRP5 (sense, 5′-GGA TAA CTT CTC AGT GGG-3′; antisense, 5′-GGA ATG GCA ATG CTC TAA AG-3′); BCRP (sense, 5′-TTA GGA TTG AAG CCA AAG G-3′; antisense, 5′-TAG GCA ATT GTG AGG AAA ATA-3′) and β2-microglubulin (sense, 5′-CCA GCA GAG AAT GGA AAG TC-3′; antisense, 5′-GAT GCT GCT TAC ATG TCT CG-3′). The PCR products, along with a 100-bp DNA ladder, were analysed by electrophoresis on agarose gels containing ethidium bromide.

#### IGF protein expression by OVCAR3 cells

The presence of proteins belonging to IGF family in OVCAR3 cells was revealed by immunocytochemistry. OVACR3 were seeded at 20,000 cells/well in glass bottom chamber slides (Lab Tek, Nunc, Naperville, IL, USA). The cells were then permeabilized and incubated for 2 h at room temperature with either specific primary antibodies (dilution 1/200) anti-IGF-I, -II, -IR or -IIR (R&D Systems, Minneapolis, MN, USA). After several washes, cells were incubated successively with biotinylated secondary antibody and streptavidine coupled to fluorescein isothiocyanate (dilution 1/500), for 45 min. Isotypic controls were performed concurrently and nuclei were DAPI-labeled. The cells were then visualized by fluorescence microscopy.

#### Analysis by gene array of gene expression in OVCAR3 cells incubated in the Hospicell supernatant

Expression of several genes was analysed by gene microarray (PIQOR Microarray; Miltenyi Biotec GmbH, Bergisch Gladbach, Germany) in OVCAR3 cells incubated in the Hospicell supernatant for 8 h at 37°C. OVCAR3 cells (5×10^6^) were lysed with TRIzol^®^ reagent (Invitrogen, Carlsbad, CA, USA) and sent to Miltenyi Biotec GmbH in dry ice. The gene array experiment was performed according to the protocol of Ducros *et al*(16). Expression of IGF-I, IGF-II, IGF-IR, IGF-IIR and IGFBP 1, 2, 3, 4, 6, 10 genes was also studied by gene array.

#### Quantitative analysis by protein array of IGF family proteins secreted by M16 cells

We analysed the supernatant of M16 cells to measure the amount of IGF family proteins (IGF-I, IGF-II, IGF-IR and IGF-IIR) using protein array technology (Human Cytokine Antibody Array, Ray Biotech, Norcross, GA). This technique is based on the principle of ELISA. Membranes on which 174 anti-cytokines were fixed with appropriate controls were used. The M16 cell supernatant, contained proteins that were linked with their specific antibodies attached to the membrane. The membrane was saturated for 2 h with BSA at room temperature to block non-specific sites. Then, the M16 cell supernatant was incubated overnight at 4°C with antibodies specific for IGF-I, IGF-II and IGF-IR. After several washes, the membranes were incubated with a mixture of biotinylated secondary antibodies overnight at 4°C. Streptavidin coupled to HRP was added to the membranes, while being incubated for 2 h at room temperature.

The presence of a protein coupled to the antibody was revealed using the ECL reagent membrane (Enhanced Chemiluminescence, GE Healthcare Europe, Saclay, France). The membrane was dried and then exposed on a photographic film (Amersham Biosciences, Montigny-le-Bretonneux, France) before being developed (Kodak X-OMAT Processor 1000). The ImageJ software (US National Institutes of Health, Bethesda, USA) was used to quantify the intensity of the spot and comparison to control experiments.

#### Studies of drug-resistance of OVCAR3-GFP cells in the presence of Hospicells

The Hospicells were seeded first at 60% confluency in a 96-well flat-bottomed cell culture plate in the presence of complete medium containing 10% FBS. After 18-h incubation, the OVCAR3-GFP cells (10,000 cells/well) were added and co-cultured for 24 h with Hospicells in the presence of IGF-I (200 ng/ml) or IGF-IR inhibitors: (PPP 1 μM) or (siRNA, 10 nM- HiPerFect, Qiagen, Courtaboeuf, France) before addition of 22.2 μM carboplatin or 1.4 μM paclitaxol (17). The effect of these cytotoxic agents was evaluated quantitatively using a GFP fluorescence reader (Wallac, PerkinElmer, Waltham, MA, USA).

#### Cell viability (MTT assay)

The same experiment was also performed on OVCAR3 cells incubated in the presence of supernatant of Hospicells as described above. The number of living OVCAR3 cells was evaluated by the MTT [3-(4, 5-dimethyl-2-thiazolyl)-2,5-diphenyl-2H-tetrazolium bromide] (Sigma-Aldrich, Saint Exupéry, France) assay after addition of 20 μl of MTT (5 mg/ml) to each well for a further 4 h incubation.

The purple precipitate was dissolved in 200 μl DMSO and the optical density was measured using a multiwell plate reader (Wallac, PerkinElmer). Cell viability was evaluated as the ratio of the optical density. Each condition was repeated in three wells and the results are expressed as the means of the three wells. The result is representative of three independent experiments.

#### Statistical analysis

The results are presented as mean ± SE and data were analysed using Student’s t-test. P-value <0.05 was considered significant.

## Results

### Effect of conditioned medium from Hospicells on OVCAR3 cells

The aim of this experiment was to study the effect of conditional medium from Hospicells on the regulation of selected genes in OVCAR3 cells *in vitro* by gene array. The major genes up or downregulated after interaction of soluble factors secreted by Hospicells on ovarian cancer cells are presented in Table I. Several genes including those for cytokine and cytokine receptors such as PDGFB, TNFSF5, VEGF-B, GPR17, gpl 130 (IL6 ST), IGFBP10 and ABCC10 (MRP7), are upregulated. Curiously, the amount of several genes encoding for transcription factors such as RASA4, STAT2, STAT6, IRF4 and MARK3 were found increased. In contrast, the expression of IL2, IL13, PAR3, ABCB3 (MDR3) were decreased. In a parallel study using code genes for all up regulated mRNAs in a database for annotation, visualization and integrated discovery (DAVID) v6.7 analysis, JAK-STAT signaling pathway was suggested.

### Expression of IGF related proteins by Hospicells

**Figure 1:**
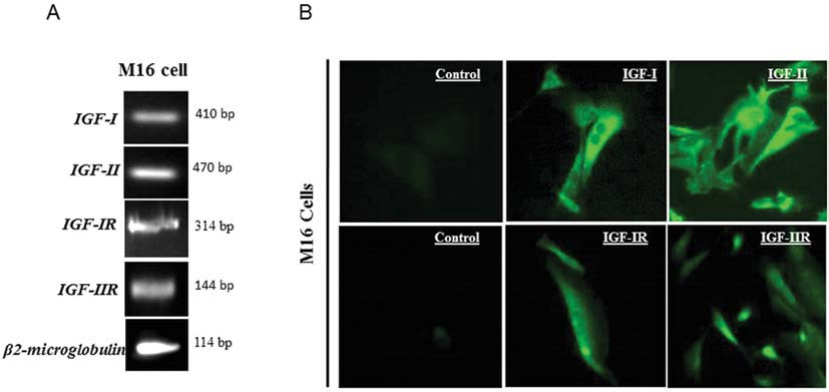
Expression of IGF components in Hospicells (M16 cells). (A) The mRNA expression: IGF-I, IGF-II, IGF-IR and IGF-IIR were analysed by RT-PCR. The total RNA of M16 cells were isolated and IGF, IGF-R mRNA were transcribed into cDNA (see Materials and methods). The resulting cDNAs were amplified using primers specific for IGF-I, IGF-II, IGF-IR and IGF-IIR. The mRNA expression of β2-microglobulin served to normalise the results. (B) IGF and IGF-R proteins were detected with the use by immunocytochemistry with specific antibodies. As negative controls, M16 cells were incubated only with the secondary antibodies: anti-mouse for ligands and anti-goat antibodies for receptors.

Because of the importance of IGF signaling pathway in cancer cell proliferation and metastasis, we concentrated our study on IGF family. Gene array, protein array, RT-PCR and immunocytochemistry methods were used for detection of IGF related family in Hospicells. The results are listed in Table II. IGF-I, IGF-II, IGF-IR, IGF-IIR and IGFBP 1, 2, 3, 4, 6 and 10 were detected by gene array. The expression of all the proteins was confirmed by protein array. RT-PCR was used to detect the presence of mRNA of IGF family members (IGF-I, IGF-II, IGF-IR and IGF-IIR) in Hospicells isolated from ascites of patients with ovarian cancer and β2-microglubulin was used as a control. Fig. 1A shows that Hospicells (M16 cells) express IGF-I, IGF-II genes as well as their receptors IGF-IR, IGF-IIR. The PCR results were confirmed by immunocytochemical analysis using specific antibodies for IGFs and their receptors (Fig. 1B). These results showed that several members of IGF family were expressed by Hospicells.

### IGFs and their receptors are expressed in OVCAR3 and implicated in cell proliferation

**Figure 2:**
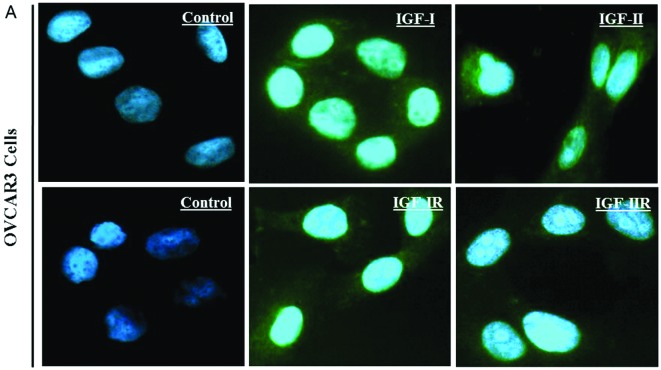

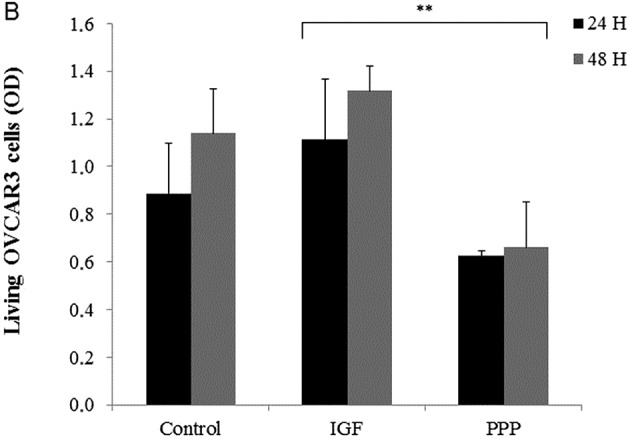
Expression of IGFs proteins in OVCAR3 cells and their effect on cell proliferation. (A) The presence of IGF-I, IGF-II, IGF-IR and IGF-IIR proteins were studied using specific antibodies and immunocytochemical methods. The negative controls were performed using only secondary antibodies. (B) Effect of IGF-I and PPP on the proliferation of OVCAR3 cells. The cells were incubated in the presence or absence of IGF-I (control) or in the presence of PPP for 24 h. The number of living OVCAR3 cells was quantified using the MTT technique.

The presence of IGF-I, IGF-II and their receptors IGF-IR and IGF-IIR proteins in ovarian cancer cell line OVCAR3 has been determined by immunofluorescence analysis using specific antibodies. IGF-I, IGF-II and IGF-IR are expressed at significant levels in OVCAR3 cells (Fig. 2A). In contrast, the immunofluorescence of the IGF-IIR was low, indicating that these cells express the receptor rather weakly. In parallel, we studied, using the MTT technique, the effect of the growth factor IGF-I on OVCAR3 cell proliferation. This experiment was performed in serum-free medium to avoid skewing the results of the study. We also investigated whether inhibition of IGF-IR with picropodophyllin (PPP) decreased OVCAR3 cell proliferation. The result (Fig. 2B) shows that OVCAR3 cells are sensitive to the effect of IGF-I and their proliferation increases at 48 h. We also found that inhibition of IGF-IR, indispensable for transmitting the effects of IGF-I in cells, led to a strong reduction in the growth and proliferation of OVCAR3 cells.

### Expression of ABC genes in OVCAR3 cells

**Figure 3:**
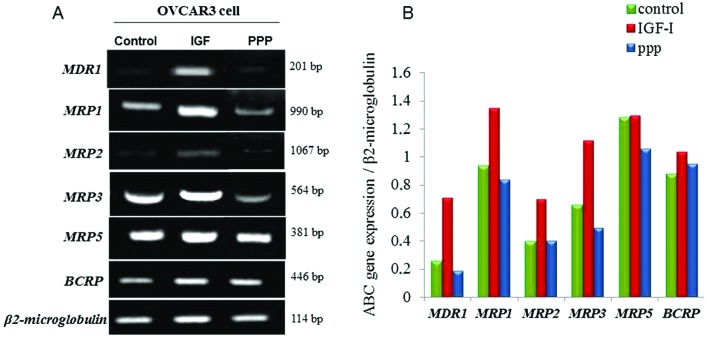
Effect of IGF-I on ABC gene expression in OVCAR3 cells. OVCAR3 cells were incubated in culture medium without (control cells), the presence of IGF-I (200 ng/ml culture medium) or in the presence of PPP (1 μM/ml) for 8 h. Expression of ABC genes regulated by IGF-I was analysed by RT-PCR. The intensity of bands of each experiment was evaluated using ImageJ software. The results were normalised with the expression of the mRNA of β2-microglobulin.

ABC transmembrane proteins play an important role in drug resistance of cancer cells because they expel from the cells the anticancer drugs. Indeed, overexpression of ABCB1 (MDR1 gene and its P-gp protein) is directly involved in the phenomenon of multidrug resistance *in vitro*. Ovarian cancer cell line OVCAR3 is characterized by an average degree of aggressiveness. We sought to determine the expression of ABC genes by OVCAR3 cells with RT-PCR using primers specific for MDR1, MRP1, MRP2, MRP3, MRP5 and BCRP (see Materials and methods). The amount of mRNA for β2-microglobulin was also analysed to normalize the results. The results in Fig. 3 show that OVCAR3 cells do not express MDR1 and MRP2 genes at a significant level. The expression of MRP1 and MRP3 is above basal levels whereas expression of MRP5 and BCRP genes are strong. The results shown (Fig. 3) present the expression of ABC genes when the OVCAR3 cells were treated with IGF-I. In the presence of the inhibitor PPP, the expression of the MDR1 gene was reduced. We obtained a similar result for the expression of MRP2. We also found that IGF-I increases the expression of MRP1 and MRP3. This expression is reduced in the presence of the inhibitor of IGF-IR when compared to control cells. In contrast, there was no significant difference in expression of MRP5 and BCRP in the presence of IGF-I or PPP compared to control cells. The intensity of bands in each experiment was evaluated using ImageJ. The results were normalised with the expression of the mRNA of β2-microglobulin.

### IGF-I regulates the expression of ABC genes in OVCAR3 cells by PI3-kinase, MEK and JAK2-STAT3 pathways

**Figure 4:**
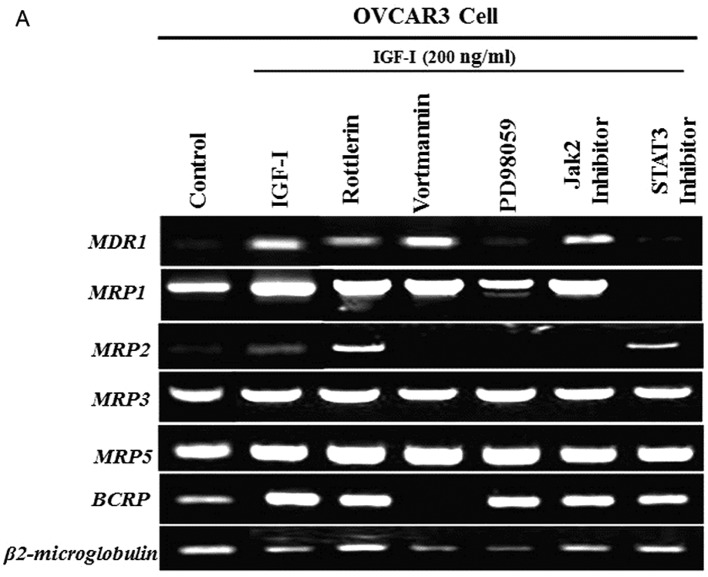

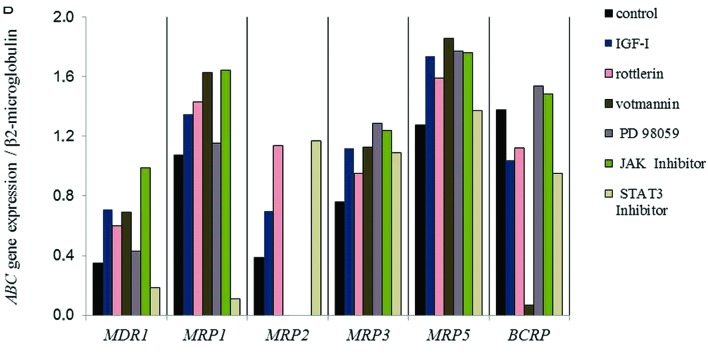
Inhibition of ABC gene expression by inhibitors of signaling pathways. (A) OVCAR3 cells were treated with IGF-I or not (control) for 8 h in a culture medium without FBS and under the same conditions as the previous experiment. Then, inhibitors rottlerin, wortmannin, PD98059, JAK2 inhibitor and STAT3 inhibitor were added to the cell medium. (B) The results were normalised with the mRNA expression of β2-microglobulin and intensity of PCR bands of each experiment was measured using ImageJ software.

To elucidate the mechanism of drug resistance facilitated by the expression of ABC genes in OVCAR3 cells in the presence of IGF-I or its absence (negative control), we tested the effects of five inhibitors of signaling pathways: rottlerin (PKC inhibitor), wortmannin (PI3 kinase inhibitor), PD98059 (MEK inhibitor), Jak2 inhibitor and STAT3 inhibitor. ABC genes expression was induced at different levels by IGF-I (Fig. 4A and B; Table III). Indeed, the inhibitor PD98059 decreased the expression of three ABC genes MDR1, MRP1 and MRP2; the wortmannin inhibits completely the expression of BCRP and MRP2 in OVCAR3 cells and Jak 2 inhibitor reduces the expression of MDR1 and MRP2. Our attention has focused on the effect of the STAT3 inhibitor on expression of MDR1 and MRP1. The expression of these genes was totally inhibited by the addition of STAT3 inhibitor. This is important since MDR1 and MRP1 are known for their role in the resistance of ovarian cancer against chemotherapy, including carboplatin and taxol. In contrast, no difference in MRP3 and MRP5 gene expression was observed in the presence of various inhibitors as compared to OVCAR3 control cells incubated only with IGF-I.

### The effect of IGF-I on drug resistance of OVCAR3 cells with conditioned medium from Hospicells

Inhibition of IGF-IR (or signaling pathways activated by this receptor) can alter ABC gene expression, mainly MDR1. The OVCAR3 cells were incubated in a serum-free medium (control cells) or with supernatants of Hospicells in the presence of IGF-I or PPP. The incubation of OVCAR3 with Hospicell supernatant was carried out in the presence (or absence) of carboplatin or taxol. The aim was to study the effect of IGF-I on the resistance of OVCAR3 cells to these chemotherapteutic agents.

**Figure 5:**
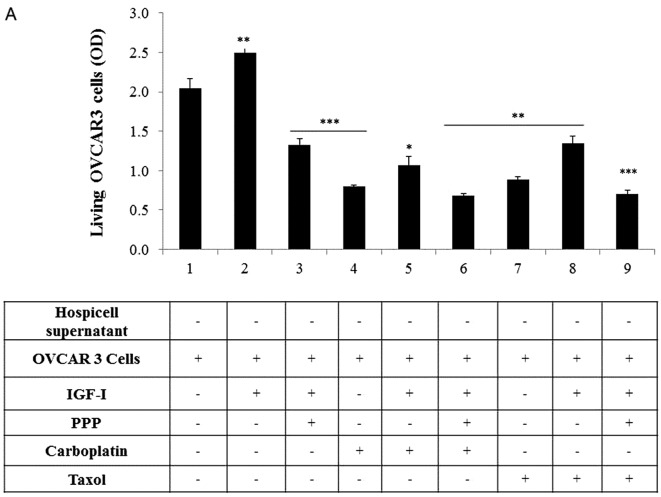

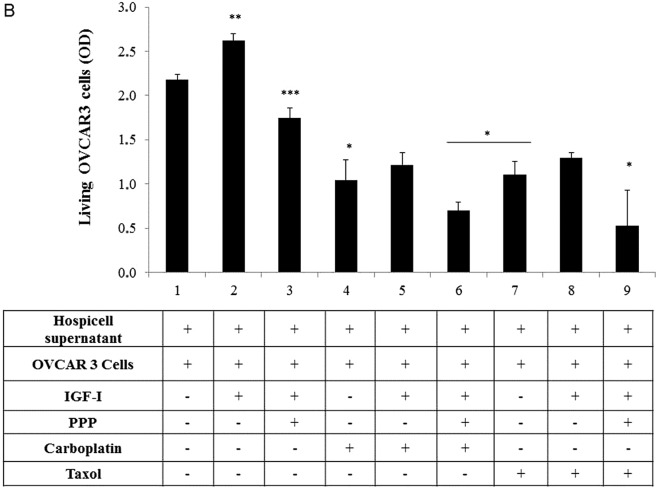
Effect of IGF-I on drug resistance of OVCAR3 cells analysed by MTT. (A) OVCAR3 cells were incubated in the presence of IGF-I (200 ng/ml) or PPP (1 μM/ml) in culture medium without FBS. After 24 h of incubation, carboplatin (22.2 μM) and taxol (1.4 μM) were added to the incubation medium of OVCAR3 cells. The number of living OVCAR3 cells was evaluated by optical density using the MTT technique. (B) OVCAR3 cells were incubated in the same conditions as the previous experiment in the presence of the Hospicell supernatant. Statistical evaluation was then given as mean ± SE (n=3), and expression difference was found to be significant (^*^P<0.05, ^***^P<0.005) compared to untreated control cells.

The results in Fig. 5A show that proliferation of OVCAR3 cells is greater in the presence of IGF-I as compared to control cells and decreases when the inhibitor PPP is present. These observations confirm our previous results shown above in Fig. 2B. The increase in proliferation of OVCAR3 cells due to the presence of IGF-I was diminished, however, when taxol or carboplatin was added.

We also found that in the presence of Hospicell supernatant (Fig. 5B), the OVCAR3 cells showed increase in proliferation compared to control cells. This proliferation was amplified in the presence of IGF-I and clearly decreased in the presence of PPP. This suggests that the Hospicell supernatant contain most probably other growth factors in addition to IGF-I that have an effect on tumor cells.

In the presence of IGF-I and Hospicell supernatant as compared to controls, a significant number of OVCAR3 cells survived, and therefore resisted the action of carboplatin or taxol action.

### Co-culture of OVCAR3-GFP cells and Hospicells in the presence of IGF-I and induction of drug resistance

**Figure 6:**
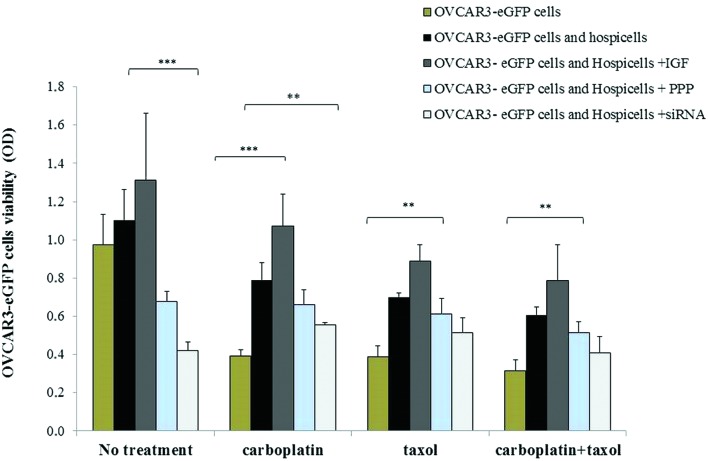
The Hospicells in the presence of IGF-I increased drug resistance of OVCAR3-eGFP cells. OVCAR3-eGFP cells were incubated under the same conditions as the previous experiment and in the presence of hospicells in culture medium without FBS. After 24 h of incubation, carboplatin (22.2 μM) and taxol (1.4 μM) were added and the number of living OVCAR3-eGFP cells was evaluated by the MTT technique. Mean ± SE (n=3), the expression difference was significant (^*^P<0.05, ^***^P<0.005).

OVCAR3 cells transfected with a fluorescent protein (GFP) were co-cultured with Hospicells in the presence or absence of IGF-I or PPP for 24 h at 37°C. An increase in OVCAR3-GFP cell proliferation was observed when co-cultured with Hospicells, compared with control cells, provided they were not exposed to the chemotherapeutic agents, carboplatin or taxol. The proliferation rate increased with addition of IGF-I and decreased in the presence of added inhibitor PPP (Fig. 6). We also used siRNA anti-IGF-IR in the same conditions and in place of PPP. Addition of siRNA instead of PPP further decreases drug resistance and therefore inhibited cell survival and proliferation (Fig. 6).

## Discussion

The tumor microenvironment is composed of endothelial cells, fibroblasts associated with cancer (FCA), adipocytes, smooth muscle cells and cells of the immune and inflammatory systems. These cells communicate with each other, as well as tumor cells, via cytokines and growth factors such as VEGF, IGF-I, FGF, and IL-8 (18). Many studies show the role of the stromal cells in the microenvironment and their involvements in drug resistance of tumor cells. In these studies, a set of stromal cells, called Hospicells (9), have been identified and purified from cell aggregates of ascites of patients with ovarian cancer. Hospicells interact with the ovarian tumor cells and play a role in chemoresistance of these cells by a trogocytosis mechanism (9). Furthermore, an *in vivo* study showed that Hospicells promoted the proliferation and angiogenesis of cells in ovarian cancer (12).

Because of the important role of stromal microenvironment in cancer cell behavior, we set to demonstrate first of all that the conditioned medium from Hospicells activate several genes (Table I) and favors JAK-STAT signaling pathway in ovarian cancer cell lines. Upregulation of MRP7 (ABCC10) and downregulation of MDR3 (ABCB3) suggests that the soluble factors secreted by cancer cell microenvironment can be involved in drug resistance in cancer cells. Expression of VEGFB confirmed previous observation of the role of Hospicells in angiogenesis (12). However, more elaborate studies are required for identifying the bioactive molecules in question. Among the molecules secreted by the tumor microenvironment, the growth factor IGF-I plays an important role in development and progression of various human cancers, and also in the inhibition of apoptosis (19). Elevated plasma concentrations of IGF-I has been linked to a high risk for several types of cancers including breast, prostate and lung cancer (20,21). In this study, using several techniques (Table II) we showed that Hospicells secrete IGFs and IGF related proteins. Hospicells were shown earlier (9) to express ATP biding cassette mRNA and proteins except MRP5.

Given the importance of the growth factor IGF-I in the interactions of tumor cells with stromal cells in the microenvironment, in the present study, we investigated a possible involvement of this growth factor in the regulation of ABC gene expression (MDR1, MRP1, MRP2, MRP3, MRP5 and BCRP) and its impact on the resistance of OVCAR3 cells in the presence of Hospicells against carboplatin or taxol. The results we obtained indicated that OVCAR3 cell line: i) expressed IGF-I and IGF-IR proteins. These results agree with the study by Gotlieb *et al*(22). ii) OVCAR3 cells do not express MDR1 and MRP2 genes at a significant level. The expression of MRP1 and MRP3 was above basal levels whereas expression of MRP5 and BCRP genes were strong. iii) IGF-I is involved in the regulation of ABC gene expression (MDR1, MRP1, MRP2 and MRP3) but not MRP5 and BCRP. iv) The expression of these ABC genes, usually regulated by IGF-I, is decreased when the IGF-IR is inhibited. v) The modulation of ABC gene expression by IGF-I is provided by STAT3, MEK, Jak2 and PI3-K signaling pathway. In contrast, vi) the expression of MRP5 gene is not regulated by signaling pathways activated by IGF-I. As such, it may be useful to consider intensifying the research on the signaling pathway of STAT3, in view of its importance in many cancers (23). Gest *et al*(24) showed that inhibition of STAT3 was a promising approach to reduce the aggressiveness of the ovarian cancer cells. Cytokines have been shown to mediate reversal of multidrug resistance by several authors (25). Moreover, Interferon-α upregulates MDR1 mRNA and P-gp expression in ovarian cancer (26), and in colon cancer, and MRP mRNA is upregulated by TNF-α (27). Interestingly, both these cytokines as well as IGF-I use JAK-STAT pathway signaling for the upregulation of MDR. On the other hand, a study indicated that PI3-K signaling is involved in BCRP expression and inhibiting of this pathway resulted in increased intracellular accumulation of chemotherapeutic drugs and cytotoxicity in tumor cells (28,29). We observed the effect of IGF-I on proliferation of the OVCAR3 cell line in serum-free conditions and protection of the cells from cytotoxic effects of carboplatin or taxol similarly to that observed with doxorubicin in MCF7 human breast cancer (30) and carboplatin in ovarian cancer (31). The previous reports (32–34) favor a partial involvement of ABC protein (MRP2) in drug resistance with platinum derived agents. It is well known that platinum derivatives act on multiple signal transduction pathways through mechanisms including glutathione regulation, reduction of drug transport and increased DNA adduct tolerance and repair. Here, we observed that increase of ABC protein expression in OVCAR3 or OVCAR3-Hospicells after incubation with IGF-I induces the resistance to carboplatin derived agents. This resistance can be produced by other mechanism such as DNA repair independent of the up-regulation of ABC proteins. We also evaluated the role of IGF-I on drug resistance acquired following the interaction of OVCAR3 cells and Hospicells. At first, our results showed that, in the absence of the drug, the very presence of Hospicells in culture medium increases the proliferative ability of ovarian cells, which can be explained by enrichment of the medium by growth factors. We observed, however, that Hospicells express the growth factor IGF-I but it secretes it only at low levels *in vitro*. This can be explained by the presence of IGFBP-3 which is highly secreted by Hospicells (results not shown) and which is capable of IGF-I sequestering. It is possible that Hospicells do not secrete metalloproteinase MMP-9 (12), which normally cleaves the complex IGF-I/IGFBP-3 and allows the release of IGF-I. The proliferative ability of ovarian cell line is significantly increased in the presence of IGF-I and decreases if IGF-IR is inhibited. Furthermore, we confirmed that contact between Hospicells and ovarian cancer cells was important for the acquisition of drug resistance by OVCAR3-GFP cells and which of course increased in the presence of IGF-I. We then added inhibitors of IGF-IR, PPP and siRNA, and observed a decrease in the drug resistance of OVCAR3-GFP cells. The decrease was pronounced with the siRNA. In this system, IGF-IR/siRNA was not a good inhibitor candidate, because of its non-specificity related to drug treatment. This suggests that IGF-I is not the only factor to influence the mechanism of drug resistance, but other factors may exist that are secreted by Hospicells, but the mode of action remains to be elucidated. A more in depth study is needed to understand the impact of interactions between Hospicells and tumor cells in the presence of other growth factors.

When there is no contact between Hospicells and OVCAR3 cells (in the presence of Hospicell supernatant), as compared to controls, OVCAR3 cells resist to treatment by carboplatin or taxol action and show poor survival. Upregulation of MRP-7, JAK-STAT signaling pathway and downregulation of MDR3 can also be implicated in these phenomena. Our results suggest that drug resistance in tumor cells is stronger when there is a synergy of effects induced by a sufficient level of IGF-I, secreted by the microenvironment, and intimate contact between Hospicells and cancer cells.

This synergy could be due to the interplay of different mechanisms. First, IGF-I, secreted by the microenvironment is involved through autocrine and paracrine action in the regulation of expression of ABC genes in OVCAR3 cells. However, we suppose that IGF-I is also involved in the regulation of ABC genes present in Hospicells. Further investigation is necessary to determine whether IGF-I actually regulates these genes, and at which level the regulation occurs need to be investigated in a future study. Other data (35) suggest that the anti-apoptotic effect of IGF-I may intervene in the decreased sensitivity to chemotherapeutic drugs *in vitro* and *in vivo*. Thus, targeting the IGF-I/IGF-IR system could serve as an approach to overcome clinical drug resistance in certain tumors. In conclusion, inhibition of IGF-IR and modulation of JAK-STAT signaling pathway can be an approach worth considering in the therapy of ovarian cancer.

## Figures and Tables

**Table I:** Up and downregulation of the gene activation after treatment of OVCAR3 cells by conditional medium from Hospicells *in vitro*.

Genes positively regulated		Genes negatively regulated	
	
Gene reference	Gene name	Gene reference	Gene name
NM-002608	PDGFB (platelet derived growth factor B)	NM-002188	IL-13 (interleukin 13 precursor)
NM-000074	TNFSFB (tumor necrosis factor ligand superfamily member 5)	NM-000586	IL-2 (interleukin 2 precursor)
NM-003377	VEGFB (vascular endothelial growth factor B)	NM-004101	PAR3 (proteinase activated receptor 3 precursor)
NM-005291	GPR-17 (putative G protein coupled receptor)	NM-000544	ABCB3 (ATP binding cassette B3)
NM-002184	IL6ST (interleukin 6 signal transducer)		
NM-006989	RASA4 (RAS GTPASE activating protein 4)		
NM-005419	STAT2 (signal transducer and activator of transcription 2)		
NM-003153	STAT6 (signal transducer and activator of transcription 6)		
NM-000629	INFAR1 (interferon alpha receptor 1)		
NM-002460	IRF4 (interferon regulatory factor 4)		
NM-002376	MARK3 (MAp/microtubule affinity regulating kinase 3)		
NM-001554	IGFBp10 (insulin like growth factor binding protein 5 precursor)		
NM-033450	ABCC10 (ATP binding cassette, C10)		

**Table II:** Expression of IGF related proteins by Hospicells using different methods such as gene array, protein array, RT-PCR analysis and immunocytochemistry.

IGF protein family	Gene array	Protein array	RT-PCR	IF
IGF-I	+	+	+	+
IGF-II	+	+	+	+
IGF-IR	+	+	+	+
IGF-IIR	+	+	+	+
IGFBP-1	+	+	Nt	Nt
IGFBP-2	+	+	Nt	Nt
IGFBP-3	+	+	Nt	Nt
IGFBP-4	+	+	Nt	Nt
IGFBP-6	+	+	Nt	Nt
IGFBP-10	+	Nt	Nt	Nt

Nt, not tested.

**Table III:** Corresponding inhibitors for ABC gene expression in ovarian cell line treated by IGF-I.

Inhibitors used	ABC gene expression
Rottlerin (PKC inhibitor)	-
Wortmannin (PI3 kinase inhibitor)	BCRP and MRP2
PD98059 (MEK inhibitor)	MDR1, MRP1 and MRP2
Jak2 inhibitor	MDR1 and MRP2
STAT3 inhibitor	MDR1 and MRP1
